# Therapist-Guided Forest Therapy in Adolescents: Operational Feasibility and Anxiety-Symptom Signals from a Two-Classroom Quasi-Experimental Pilot Study

**DOI:** 10.3390/bs16091647

**Published:** 2026-09-14

**Authors:** Francesco Meneguzzo, Federica Zabini

**Affiliations:** 1 Institute of Bioeconomy, National Research Council, 10 Via Madonna del Piano, I-50019 Sesto Fiorentino, Italy; federica.zabini@cnr.it; 2 Consorzio LaMMA, Via Madonna del Piano 10, I-50019 Sesto Fiorentino, Italy

**Keywords:** forest therapy, adolescents, anxiety symptoms, acute mood, school-based prevention, nature-based intervention

## Abstract

Adolescence is a sensitive period for anxiety symptoms, yet evidence on repeated forest therapy in secondary-school populations remains limited. This two-classroom quasi-experimental pilot examined operational delivery, acute affective responses, and follow-up anxiety-symptom signals after four therapist-guided forest therapy sessions in a coastal pine forest. Two intact, non-clinically selected classrooms were allocated without randomization. The principal all-classroom-assigned analysis included 26 intervention-classroom and 28 usual-activities control-classroom students aged 17–18 years. The sole principal outcome domain was the Spence Children’s Anxiety Scale (SCAS) total raw score, measured at baseline and four follow-ups through 10–11 weeks; acute mood was assessed with the Profile of Mood States—Adolescents (POMS-A) before and after each session. Descriptively larger SCAS reductions occurred in the intervention classroom at every follow-up. The largest baseline-adjusted difference occurred 8–9 weeks post-intervention (coefficient = −5.14 points, 95% confidence interval [−10.25, −0.04]; two-sided *p* = 0.048; false-discovery-rate-adjusted q = 0.135), and no principal SCAS comparison survived multiplicity correction. Acute total mood disturbance improved most clearly during the third scheduled session (*n* = 21; mean improvement = 9.71; q = 0.002; Cohen’s dz = 1.29), but POMS-A had no control-classroom comparator. Discordant exploratory findings included lower adjusted conduct/behavior (q = 0.028) and lower post-intervention connectedness to nature (q = 0.005) in the intervention classroom. Because condition was inseparable from classroom and no active comparator was included, favorable and unfavorable between-classroom differences cannot be attributed to forest therapy and do not establish efficacy. The study documents delivery in this setting and provides descriptive signals and design information for preregistered, adequately replicated cluster-randomized research.

## 1. Introduction

Adolescence is characterized by rapid biological, cognitive, emotional, and social change (Blakemore, 2019). The integration of these changes into a coherent sense of identity is a central developmental task (Sharp & Wall, 2018). This transition is also a period of heightened vulnerability to mental-health problems and the onset of lifetime disorders (Guthold et al., 2023). In a large-scale meta-analysis of 192 epidemiological studies, the peak and median ages at onset for any mental disorder were 14.5 and 18 years, respectively, while anxiety- and fear-related disorders showed a median age at onset of 17 years (Solmi et al., 2022). These observations support early preventive approaches during mid-to-late adolescence, with schools offering an accessible setting for low-risk and contextually acceptable interventions.

Recent cohorts of adolescents appear to experience higher rates of depression, anxiety, and self-harm than earlier generations (Borg et al., 2025). Evidence also suggests that the COVID-19 pandemic intensified pre-existing upward trends in youth depression and anxiety rather than creating them de novo (Bosmans et al., 2025). Approximately one in seven individuals aged 10–19 years experiences a mental disorder, and anxiety disorders are among the most prevalent conditions in this age group, affecting an estimated 4.1% of 10–14 year olds and 5.3% of 15–19 year olds (World Health Organization, 2025).

Given this burden and the persistence of childhood and adolescent mental-health problems into adulthood, evidence-based preventive programs have been recommended for integration into school practice (Baffsky et al., 2023; Mulraney et al., 2021).

At the same time, adolescents may have increasingly limited direct contact with nature (Dong & Geng, 2023). Urbanization, lifestyle change, and the pervasive use of screens and social media have contributed to declining outdoor activity (Edwards & Larson, 2020). Nature-based approaches have therefore attracted attention as potential supports for affect regulation, attentional restoration, and psychological well-being alongside established clinical and educational strategies (Moll et al., 2022; Saeedy Robat et al., 2026).

Contact with natural environments has been associated with mental-health and cognitive benefits across age groups (Bratman et al., 2019). Greater surrounding greenspace at home and school has been associated with lower anxiety symptoms in children (de la Osa et al., 2024), while reviews indicate that nature exposure can influence nervous-system activity, attention, and cognitive resource allocation in children and adolescents (Liu et al., 2025; Nguyen & Walters, 2024; Quintela Do Carmo et al., 2025). However, a large longitudinal study of university students suggested that quantified outdoor exposure alone may be insufficient: experiential engagement, mindful attention, and perceived connection to nature appeared more closely related to changes in anxiety (Bloomfield et al., 2026).

A recent second-order meta-analysis found that nature-based interventions reduced stress, anxiety, and depression across populations but also identified a marked lack of rigorous adolescent studies and the frequent use of passive control conditions (Saeedy Robat et al., 2026). A systematic review of European nature-based solutions likewise found that most studies involved adults and called for more adolescent-specific, longitudinal, and experimental research with a clearer characterization of intervention components (Chakravorty et al., 2026).

Among nature-based interventions, forest therapy is generally understood as a structured, guided, multisensory, and slow-paced immersion in a forest environment. Reviews report short-term improvements in stress, depressive symptoms, anxiety symptoms, and mood, although study quality and intervention protocols remain heterogeneous (Chen et al., 2025; Qin et al., 2026; Zhang et al., 2023). In adults, a comparison of therapist-guided and otherwise comparable self-guided forest immersion reported larger short-term improvements with guidance in state anxiety, total mood disturbance, and self-esteem (Rivieccio et al., 2025). That study addressed delivery mode and immediate outcomes; it did not establish whether repeated sessions produce longer-lasting changes, particularly in adolescents.

Because terminology overlaps, we use forest therapy here to denote the complete structured program evaluated in this study: facilitator-guided, slow and low-exertion movement in a forest with repeated multisensory-attention invitations. Forest bathing is used more broadly for intentional sensory engagement with a forest and may be guided or self-guided; nature exposure and outdoor activity are broader categories. Although delivered by school mental-health professionals, the present program was an authorized educational and preventive activity, not individual or group psychotherapy conducted outdoors and not treatment for a diagnosed disorder. Forest setting, professional guidance, sensory prompts, movement, peer-group participation, expectancy, and time away from lessons occurred together; the design cannot identify an active ingredient.

The emerging but still limited literature has examined forest therapy and forest bathing in adolescent or youth populations. Structured programs have been associated with improvements in well-being, self-esteem, resilience, and nature connectedness among adolescents with mental-health disorders, juveniles under protective detention, and students participating in urban forest-bathing activities (Kil et al., 2023; Kim et al., 2020; McEwan et al., 2022). However, these studies often involved selected populations, uncontrolled pre–post designs, short follow-ups, or outcomes other than anxiety symptoms in ordinary school classrooms.

Nature-based interventions may also influence connectedness to nature, with potential implications for well-being, pro-environmental behavior, and social relationships (Madera et al., 2025). Nevertheless, connectedness to nature should be distinguished from clinical or symptom outcomes and requires baseline measurement if it is to be evaluated as a mediator of intervention effects.

A central unresolved question is whether acute affective responses during individual nature-based sessions track anxiety-symptom trajectories over subsequent weeks. Candidate proximal pathways for transient mood change include temporary disengagement from school demands, guided sensory attention, low-intensity movement, changes in arousal or perceived stress, and group co-regulation. Persistent anxiety-symptom change would require any relevant process to endure or generalize beyond individual sessions and across daily life. None of these candidate pathways was measured here. Acute and longitudinal outcomes may therefore differ in magnitude, timing, determinants, and evidentiary meaning, and their relationship must be tested directly rather than inferred.

The present study investigated a repeated forest therapy program in a secondary-school population and explored dose-, repetition-, and duration-related patterns, which remain important gaps in the translation of outdoor therapies into mainstream mental-health practice (Buckley et al., 2018).

This study was designed as an operational-feasibility and descriptive signal-detection pilot, not as an evaluation of treatment efficacy. The SCAS total raw score across the four scheduled follow-ups was the sole principal outcome domain; no single follow-up was retrospectively designated as a primary endpoint. The directional scientific hypothesis was that the intervention classroom would show larger baseline-to-follow-up SCAS reductions than the usual-activities classroom, but all inference was two-sided because the pilot was not preregistered. For reporting, the all-classroom-assigned, baseline-adjusted analysis was principal, and the attendance-restricted analysis was a sensitivity analysis. Acute POMS-TMD was a secondary within-intervention-classroom descriptive outcome. POMS-A subscales, baseline-threshold analyses, acute–longitudinal correlations, school outcomes, post-intervention nature-questionnaire measures, and student-level longitudinal descriptions are exploratory or hypothesis-generating. A joint characterization of acute affects and repeated longer-term anxiety-symptom assessments—without treating one as a surrogate for the other—is the principal conceptual contribution.

## 2. Materials and Methods

### 2.1. Study Design, Participants and Setting

The study initially involved 54 students from two fourth-year classes following the same Human Sciences high-school curriculum at a public school in Cecina, Tuscany, Italy. The project was embedded in an authorized school-to-work training program focused on forest therapy and psychological well-being and was implemented with school and local public-health support. Institutional authorization, informed consent, and data-protection procedures are reported in the back-matter statements. The classrooms followed the same curriculum and shared most teachers. Before data collection, students and teachers attended a 2 h plenary meeting on the study rationale, procedures, and schedule. The school psychotherapist and psychologist who delivered the intervention received protocol training from the research team, based on a previously published therapist-guided forest-immersion procedure (Rivieccio et al., 2025). The same school psychotherapist and school psychologist delivered all four sessions.

The study design included one intervention classroom (class 4AU), which received the forest therapy program during school hours, and one control classroom (class 4BU), which continued usual school activities. Participants were allocated by classroom: 26 students in the intervention classroom and 28 in the control classroom. Students were not screened or selected on the basis of anxiety symptoms, psychiatric diagnosis, or treatment status; the sample therefore represents two intact, non-clinically selected classrooms rather than a clinical anxiety cohort.

With only one classroom per condition, classroom and intervention condition were perfectly confounded, so no classroom-independent intervention effect could be estimated. The research dataset did not include psychiatric diagnoses, depressive-symptom measures, learning-disability or neurodevelopmental status, medication, psychotherapy, or other concurrent-treatment variables.

Classroom-level allocation was used because the study was embedded in an educational program and had to preserve ordinary school organization. Baseline comparability was assessed descriptively for age and gender and statistically for baseline SCAS scores, as reported in Section 3.1. No formal a priori power analysis was conducted; the sample size was determined by the two intact classrooms participating in the authorized school-to-work program, consistent with the pilot quasi-experimental design. The intervention covered four consecutive weekly sessions in March 2026. The forest therapy sessions were held in the “Tomboli di Cecina” Biogenetic Nature Reserve, a protected coastal pine forest near Marina di Cecina, Tuscany, Italy. The reserve extends for approximately 15 km along the Tyrrhenian coast, north and south of the Cecina River mouth, on low-elevation sandy dune and back-dune systems. Vegetation is dominated by stone pine (*Pinus pinea*), with Mediterranean scrub, junipers, holm oak (*Quercus ilex*), and other coastal species distributed along the sea–inland ecological gradient. Sessions were conducted on a flat, accessible dirt path within the pine forest, so that the protocol could emphasize sensory attention, slow walking, and therapist-guided immersion while minimizing physical exertion.

**Figure 1:**
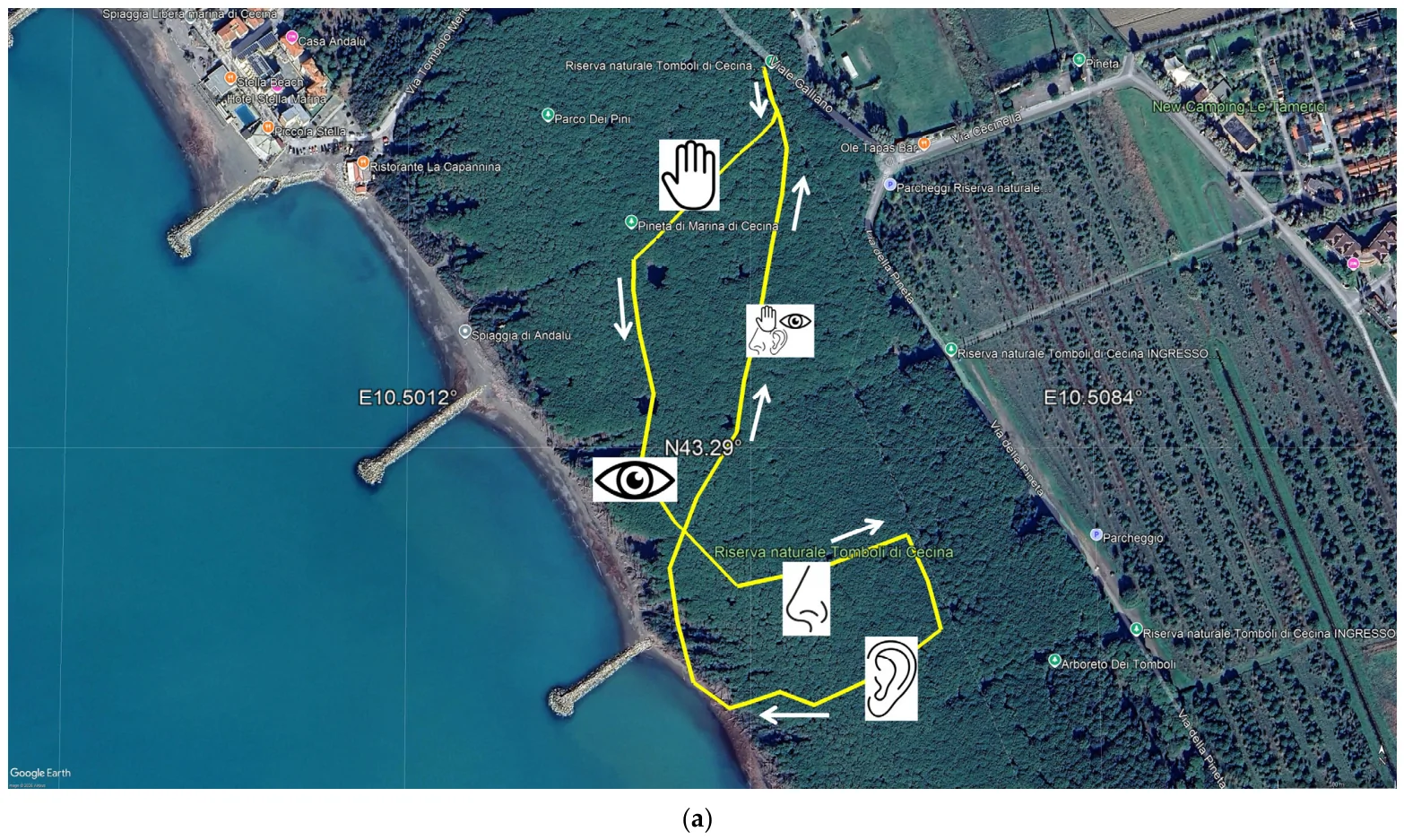

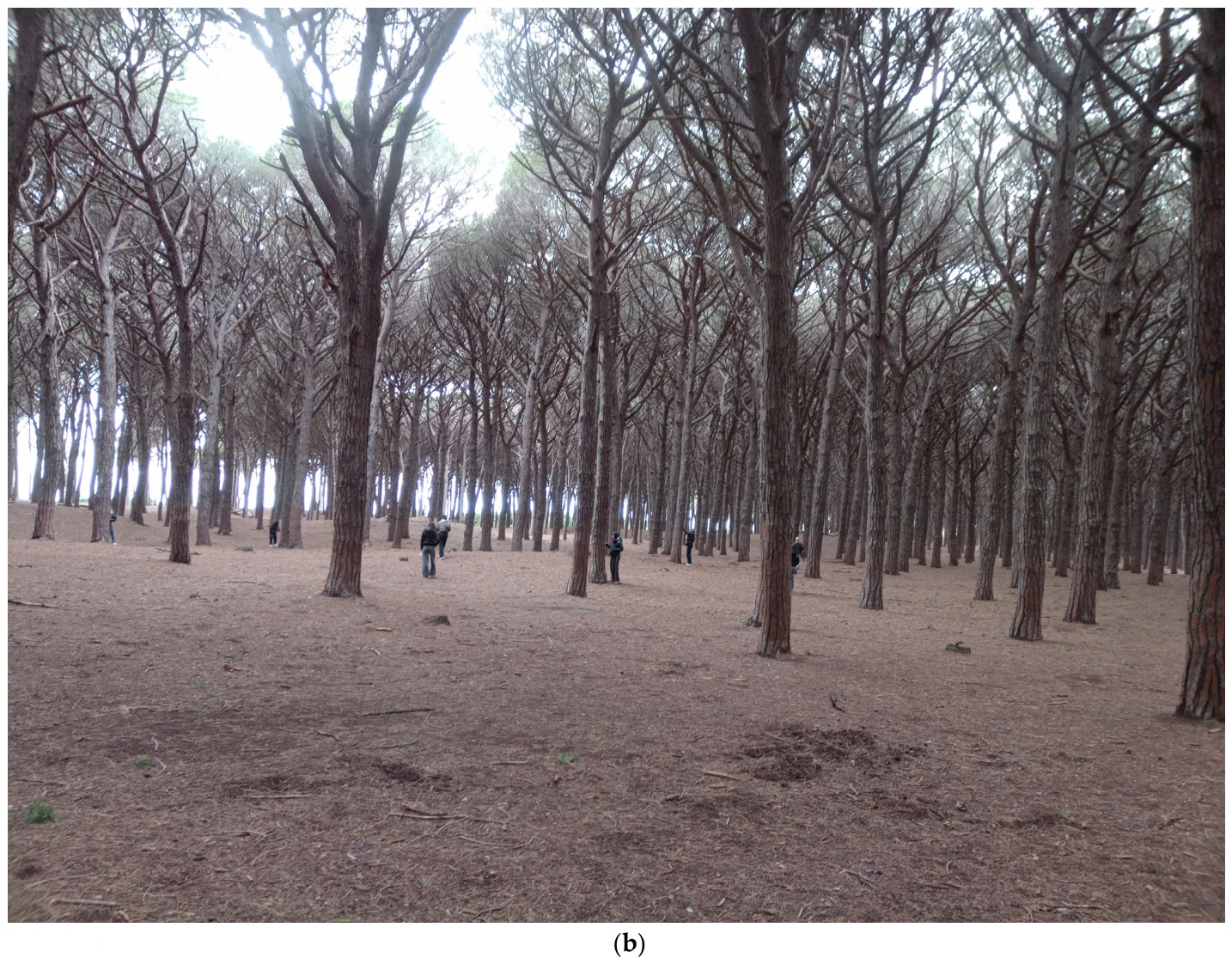
Forest therapy setting. (**a**) The yellow line and white arrows indicate the route and direction of travel. Black-and-white symbols identify stops for sight (eye), hearing (ear), touch (hand), smell (nose), and multisensory integration (combined symbols). Other colored icons are background-map points of interest, not study markers. Italian place names are retained; descriptive terms include riserva naturale (nature reserve), pineta (pine forest), spiaggia (beach), parcheggio/parcheggi (parking), and ingresso (entrance). The background satellite image was exported from Google Earth Pro (version 7.3.7.1155, 64-bit); the embedded attributions “Google Earth” and “Image © 2026 Airbus” are retained. The route, directional arrows, and sensory-stop symbols were added by the authors. (**b**) Photograph taken during a forest therapy session (photo: F. Meneguzzo).

Figure 1 shows the forest therapy route and the forest setting. The approximately 1.5 km route was flat and located at 4 ± 3 m above sea level.

Each session lasted approximately 3 h during school time and was led by a school psychotherapist, supported by a school psychologist. Sessions were held in the morning, approximately from 09:00 to 12:00 local standard time. The intervention followed a structured therapist-guided format adapted from a previously published forest-immersion protocol (Rivieccio et al., 2025). It combined low-intensity slow walking with repeated stops directing attention to visual, auditory, tactile, olfactory, and multisensory aspects of the forest. The planned route, approximate duration, low physical intensity, core sensory domains, and general procedural sequence were kept as consistent as practicable, while individual prompts could be adapted to immediate group and environmental conditions. No independent observer, scored fidelity checklist, audio/video recording, session-level adherence score, or inter-rater fidelity assessment was used; fidelity therefore cannot be formally quantified. The program bundled forest exposure, slow movement, therapist guidance, sensory activities, participation with familiar classmates, and time away from ordinary lessons; the design did not isolate the contribution of any component.

### 2.2. Measures

#### 2.2.1. Persistent Anxiety Symptoms: Spence Children’s Anxiety Scale

Anxiety symptoms were assessed using the self-report Spence Children’s Anxiety Scale—Child version (SCAS-C) (Spence, 1997, 1998; Spence et al., 2003). The measure has been used with children and adolescents, including young-adolescent samples (Spence, 1998), and has been evaluated in European adolescent samples (Essau et al., 2011), while Italian studies have examined SCAS measures in different age groups and reporting formats (De Caro et al., 2025; Delvecchio et al., 2010). Published studies report good internal consistency and test–retest reliability (Delvecchio et al., 2015). The SCAS-C was selected a priori because all participants were minors at enrollment and because it assesses multiple anxiety-symptom domains. The large cited European validation sample included adolescents aged 12–17 years, whereas the present participants were aged 17–18 years; the oldest students were therefore at or just beyond the upper age represented in that evidence. Raw total scores were treated only as continuous symptom indicators. No age-standardized norms, clinical cutoffs, diagnostic classifications, or anxiety-disorder eligibility criteria were applied. The SCAS-C contains 38 anxiety-symptom items rated from never (0) to always (3); total raw scores range from 0 to 114, with higher scores indicating greater anxiety-symptom severity.

The SCAS-C also includes six positively worded filler items. These items balance the emotional tone of the questionnaire and reduce an exclusive focus on anxiety-related content, but they are not part of the standard anxiety total or subscale scores. Consistent with the original scoring method and subsequent psychometric studies, the six filler items were excluded from score calculation and analysis (Essau et al., 2011; Spence, 1998; Spence et al., 2003).

The Italian-language SCAS was administered in the classroom at baseline (SCAS1, 25 February 2026), 1–2 weeks after the final forest therapy session (SCAS2, 31 March–8 April 2026), 5–6 weeks after the final session (SCAS3, 29 April–4 May 2026), 8–9 weeks after the final session (SCAS4, 22–25 May 2026), and 10–11 weeks after the final session (SCAS5, 4–12 June 2026). All intervals are relative to the final session on 26 March 2026.

#### 2.2.2. Acute Mood States: POMS-A

Acute mood states were assessed using the 24-item adolescent Profile of Mood States (POMS-A) (Terry et al., 1999, 2003). The Italian-language questionnaire was administered only in the intervention classroom, immediately before and after each forest therapy session at the forest site. POMS-A was developed for adolescents from the abbreviated Profile of Mood States used in sport psychology (Grove & Prapavessis, 1992) and has been applied to assess acute psychological outcomes of forest therapy (Shang et al., 2025).

POMS-A yields six mood dimensions: anger, confusion, depression, fatigue, tension, and vigor; the POMS-A tension subscale was coded as ANX in the analysis dataset. Each item was rated on a five-point scale from 0 = not at all to 4 = extremely, referring to the participant’s current state. Total mood disturbance (TMD) was calculated as the sum of the five negative subscales minus vigor; higher TMD values indicate greater mood disturbance. For change analyses, positive pre-minus-post TMD values indicate improvement.

**Figure 2:**
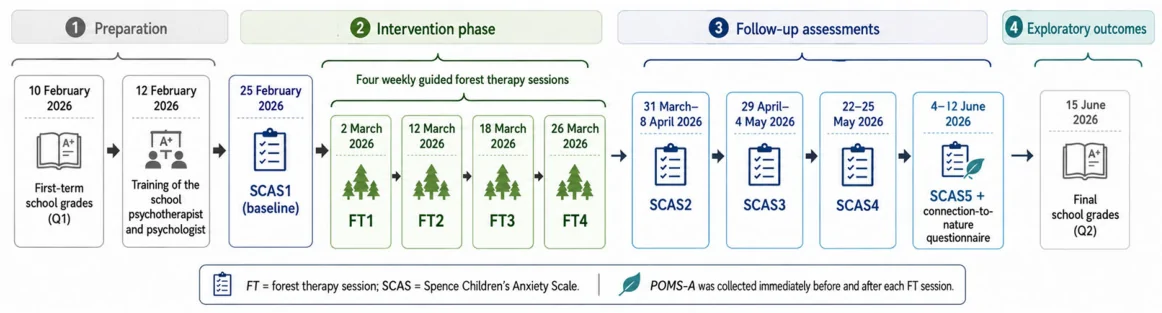
Study timeline and milestones. Forest therapy school-based intervention in two 4th-year high-school classes (intervention, 4AU; control, 4BU), Cecina, Italy, 2026. Initial timeline diagram generated using the image-generation tool available within ChatGPT (selected conversational model: GPT-5.5; OpenAI, Inc., San Francisco, CA, USA) and subsequently reviewed and edited by the authors for accuracy and completeness.

Figure 2 shows a scheme of the actions performed during the study period.

#### 2.2.3. Meteorological and Contextual Variables

For each forest therapy session, basic meteorological information was recorded on the same day from LaMMA consortium real-time data (https://www.lamma.toscana.it/meteo/osservazioni-e-dati/temperature-tempo-reale, accessed on 8 September 2026), including temperature, relative humidity, rain, and wind. Cloudiness was visually estimated by an expert meteorologist, while the discomfort index (*DI*) was calculated using Thom’s formula (Song et al., 2025), as per Equation (1):

(1) DI=T−0.55·(1−RH/100)·(T−14.5)

where *T* denotes air temperature (°C), and *RH* represents relative humidity (%). Thermal conditions were defined as follows: cold (*DI* ≤ 15), thermally neutral (16–24), and warm-hot (*DI* ≥ 25). Table 1 shows the weather and discomfort data for each forest therapy session.

These variables were used descriptively only, because only four sessions were available, and calendar session number, repeated exposure, and weather were necessarily confounded.

#### 2.2.4. Exploratory Academic Performance and Conduct Outcomes

School-performance data were obtained from official school records for the first and second grading periods of the 2025–2026 school year. First-period grades were recorded before the intervention, whereas final second-period grades were recorded after completion of the intervention and follow-up assessments. These data were used only as exploratory contextual outcomes to examine whether changes in anxiety symptoms were accompanied by parallel changes in school performance.

For each student, mean academic performance was summarized across 12 subjects that were common to both grading periods: Italian, Latin, human sciences, mathematics, civic education, motor sciences, history, philosophy, physics, art history, English, and natural sciences. Religion was excluded from the academic-performance index because of its limited comparability with curricular achievement. Conduct/behavior was analyzed separately, because it reflects teachers’ global evaluation of classroom behavior and social conduct rather than subject-specific academic achievement.

Three academic indices were computed: (i) a 12-subject mean academic score; (ii) a humanities mean score, including Italian, Latin, human sciences, history, philosophy, art history, and English; and (iii) a STEM mean score, including mathematics, physics, and natural sciences. For each index, first-period, second-period, and change scores were calculated, with change defined as second period minus first period. Positive change values therefore indicate improvement from the first to the second grading period. Because these outcomes were not part of the original primary endpoint and were potentially influenced by multiple educational and contextual factors, all school-performance analyses were considered exploratory.

#### 2.2.5. Cross-Sectional Post-Intervention Nature-Exposure and Connectedness Questionnaire

During the final follow-up period, students completed an exploratory coded questionnaire concerning their relationship with natural environments. The exposure questions referred to April–May 2026, the period following the forest therapy sessions conducted in March. Students used the same study code employed for the other measures; no direct identifiers were entered on the questionnaire.

The questionnaire distinguished three conceptually different dimensions. The first section assessed behavioral exposure to natural environments during April–May 2026, including visit frequency, frequency of visits specifically to forests or pinewoods, typical visit duration, voluntary use of natural environments for relaxation or emotion regulation, and intentional sensory attention while outdoors. A synthetic nature-exposure index was calculated from the main quantitative exposure items and rescaled from 0 to 100, with higher scores indicating greater post-intervention exposure. This dimension was included because weekly nature contact of at least 120 min has been associated with better self-reported health and well-being across population groups (White et al., 2019).

The second section assessed connectedness to nature using the 14-item Connectedness to Nature Scale (CNS), which measures an affective and experiential sense of connection with the natural world (Mayer & Frantz, 2004). The scale has been validated in the Italian context (Lovati et al., 2023). Items were rated from 1 = strongly disagree to 5 = strongly agree. Negatively worded items expressing disconnection, human superiority, or independence of personal well-being from the natural world were reverse-scored, and the mean item score was calculated so that higher values represented stronger connectedness to nature.

The third section assessed retrospective perceived change in the students’ relationship with natural environments, comparing April–May 2026 with their usual way of experiencing nature before March 2026. Items addressed perceived awareness of the effects of natural environments on mood, sensory attention, use of nature for relaxation or well-being, desire to spend time in natural settings, communication with others about nature, and perceived positive change in the personal relationship with nature. A mean perceived-change score was computed, with higher values indicating a greater perceived positive change.

Finally, two open-ended questions asked whether students had noticed anything new in their relationship with nature and whether there was a natural place to which they had returned or wished to return. These qualitative responses were used only as contextual information and were not included in the quantitative analyses.

Because no pre-intervention nature measure was collected, the questionnaire provided only cross-sectional post-intervention descriptors. Between-classroom contrasts may reflect pre-existing differences and cannot establish intervention-related change. These measures were not used to infer mediation or pre–post change in nature connectedness.

### 2.3. Missing Data

Before participant-level scale scores were calculated, a small number of missing SCAS and POMS-A item responses within otherwise substantially completed questionnaires were imputed at the item level using the KNNImputer implementation in scikit-learn, configured with five neighbors, following the procedure used in a previous study (Rivieccio et al., 2025). Imputation was performed separately for each input questionnaire file and was restricted to questionnaire rows with fewer than 20% missing item responses. Rows with 20% or more missing responses were not imputed. Imputed values were rounded to the nearest integer and constrained to the admissible response ranges of 0–3 for SCAS and 0–4 for POMS-A. No missing assessment occasion, participant-level scale total or change score, classroom mean, or whole-sample mean was imputed as a unit; however, participant-level totals could incorporate the limited number of estimated item values. In the nature questionnaire, four missing item responses (one CNS item and three perceived-change items) were replaced by the same participant’s mean response within the corresponding questionnaire block and rounded to the applicable scale; no classroom or sample aggregate was used. Records containing nature-questionnaire imputation were flagged in the analysis dataset. All resulting SCAS totals and change scores were checked for internal consistency.

### 2.4. Statistical Analysis

SCAS change scores were calculated as baseline minus follow-up, so that positive values indicate improvement. The principal all-assigned comparison retained all 54 classroom-assigned students: 26 in the intervention classroom and 28 in the control classroom. Because allocation was nonrandomized, this is not an intention-to-treat estimate. An attendance-restricted sensitivity analysis included the 24 intervention-classroom students who attended at least three sessions and all 28 control-classroom students. Between-classroom differences in change scores were examined descriptively using Mann–Whitney U and Welch tests (Delacre et al., 2017; Fernández-Abascal & Martín-Díaz, 2021). All tests were two-sided. Two-sided 95% confidence intervals, standardized mean differences, raw *p*-values, and multiplicity-adjusted q-values were prioritized. The directional hypothesis informed the scientific rationale only and did not determine inferential tails.

For each follow-up, the principal analysis of covariance (ANCOVA) model used follow-up SCAS as the outcome, classroom as the main predictor, and baseline SCAS as a covariate (Peter, 2017). Heteroskedasticity-consistent HC3 standard errors were used because of the small sample. Baseline SCAS comparability was assessed with a two-sided Mann–Whitney U test. The four baseline-adjusted follow-up estimates were treated as one principal inferential family.

Acute POMS-A pre–post changes were analyzed within the intervention classroom using all available observations and paired Wilcoxon signed-rank tests (Mao et al., 2017), with Benjamini–Hochberg false-discovery-rate (FDR) correction (Glickman et al., 2014). All POMS-A tests were two-sided; correction was applied across the 28 scheduled-session-by-domain contrasts and, separately, the 28 attended-order-by-domain contrasts. For the principal SCAS comparisons, FDR correction was applied across the four follow-ups separately for Mann–Whitney, Welch, and HC3 ANCOVA *p*-values. Additional exploratory families comprised 21 attendance-restricted baseline-threshold tests, 16 acute–longitudinal correlations, four all-assigned academic/conduct ANCOVA models, and five all-assigned cross-sectional post-intervention nature-questionnaire comparisons. Mixed-effects, generalized-estimating-equation, responder, moderation, and threshold analyses were treated only as exploratory sensitivity descriptions and were not used as independent inferential support; none can resolve the one-classroom-per-condition confounding. Both raw *p*-values and FDR-adjusted q-values are reported, with multiplicity-adjusted interpretation based on q < 0.05. Standardized mean differences were interpreted using conventional benchmarks (Cohen, 2013). Throughout, student-level estimates describe separation between these two classrooms after measured adjustment and cannot support classroom-independent causal inference.

The dataset was initially organized in Microsoft^®^ Excel^®^ for Microsoft 365 MSO (Version 2509, Microsoft, Redmond, WA, USA). Statistical analyses were performed in Python (version 3.13) using NumPy (version 2.3.2), pandas (version 2.3.1), SciPy (version 1.16.1), statsmodels (version 0.14.6), and matplotlib (version 3.10.6). Item-level k-nearest-neighbor imputation was implemented using KNNImputer from scikit-learn (version 1.7.1).

## 3. Results

### 3.1. Participant Flow and Baseline Characteristics

The study initially involved 54 students: 26 in the intervention classroom (4AU) and 28 in the control classroom (4BU).

In the intervention classroom, 11 students attended all four sessions, 13 attended three sessions, one attended two sessions, and one attended none. All 26 were retained in the principal all-assigned analysis. The 24 students attending at least three sessions contributed to the secondary attendance-restricted sensitivity analysis.

As shown in Table 2, the principal all-assigned cohort comprised 26 students in the intervention classroom and 28 in the control classroom. It included 51 female and 3 male students (51/54; 94.4% female). Baseline SCAS total raw scores were closely comparable between classrooms (41.88 ± 15.04 vs. 42.54 ± 16.28; Cohen’s d = 0.04; Mann–Whitney *p* = 0.842). The attendance-restricted sensitivity cohort included 49 female and 3 male students (49/52; 94.2% female).

### 3.2. Main SCAS Analyses

Table 3 summarizes SCAS total raw scores and baseline-to-follow-up changes in the principal all-assigned cohort. In the intervention classroom, the mean SCAS score decreased from 41.88 at baseline to 36.65 at 1–2 weeks and 35.88 at 5–6 weeks post-intervention, then increased to 38.15 at 8–9 weeks and was 37.42 at 10–11 weeks. In the control classroom, the mean decreased from 42.54 at baseline to 40.29 and 40.32 at the first two follow-ups, increased to 43.89 at 8–9 weeks, and returned to 42.54 at 10–11 weeks. The descriptive between-classroom difference in change was largest at 8–9 weeks and smaller at the final follow-up.

As a descriptive check for a total-score ceiling, no participant attained the theoretical maximum of 114. Baseline maxima were 70 in the intervention classroom and 79 in the control classroom, and the highest score at any assessment was 89. Thus, no obvious total-score ceiling was observed; this check does not establish age-appropriate measurement validity.

**Figure 3:**
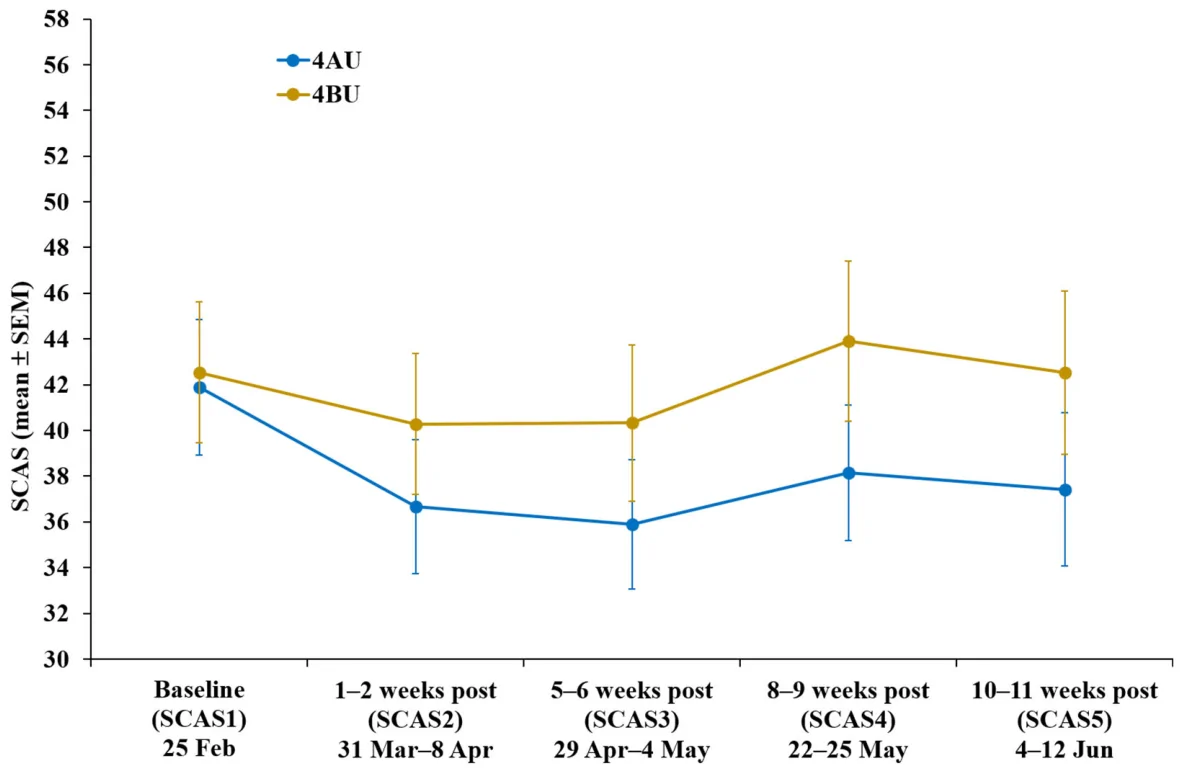
Mean SCAS trajectory by classroom in the principal all-assigned cohort (4AU *n* = 26; 4BU *n* = 28). Follow-up intervals and date ranges are relative to the final forest therapy session on 26 March 2026. Vertical bars indicate the standard error of the mean (SEM).

**Figure 4:**
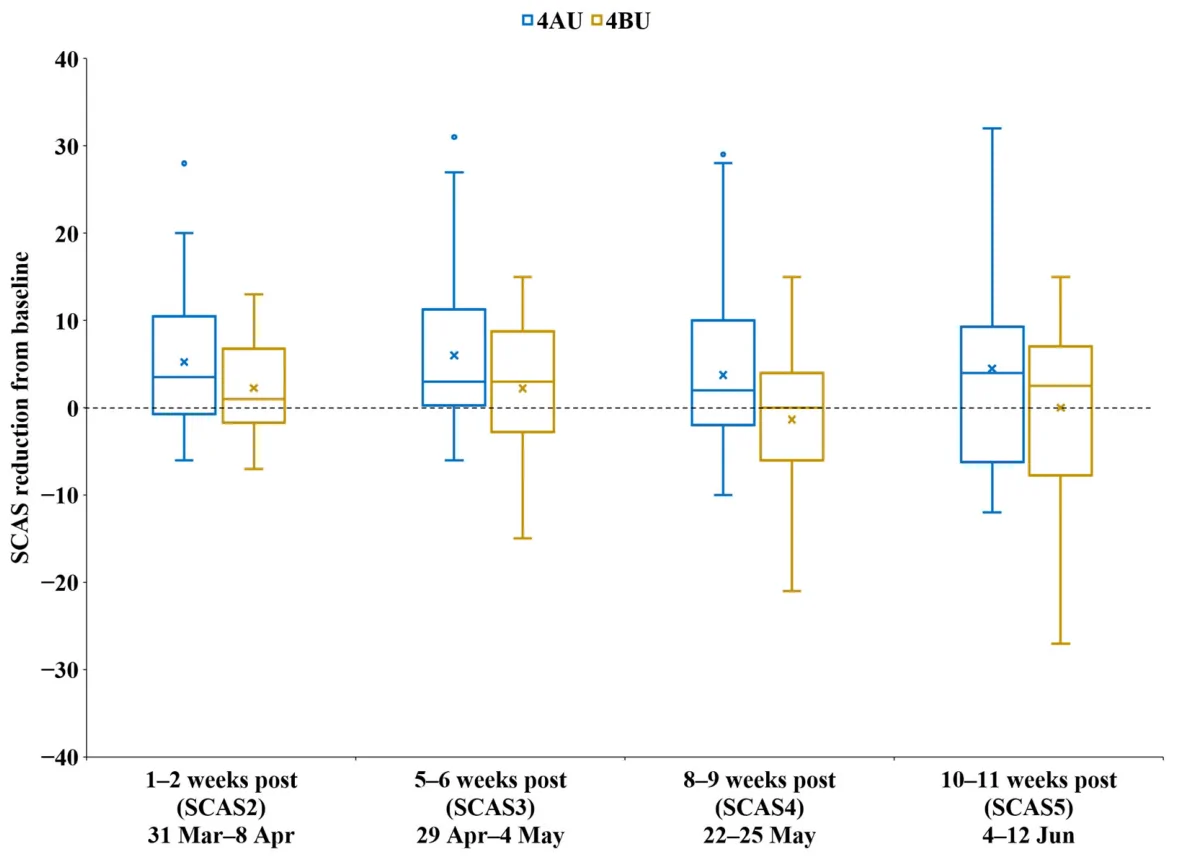
Distribution of SCAS reductions from baseline by classroom in the principal all-assigned cohort (4AU *n* = 26; 4BU *n* = 28). Follow-up intervals and date ranges are relative to the final forest therapy session on 26 March 2026. Boxes extend from the first to the third quartile (interquartile range, IQR); horizontal lines within the boxes indicate medians, and × markers indicate means. Whiskers extend to the most extreme observations within 1.5 × IQR below the first quartile or above the third quartile; points beyond the whiskers are outliers. Quartiles in this figure were calculated using Excel’s exclusive method; the IQRs in Table 3 use inclusive, linearly interpolated quartiles. Positive values indicate lower SCAS scores than at baseline.

The intervention classroom showed descriptively larger mean reductions from baseline at all four follow-ups, with substantial uncertainty and overlap between classroom distributions. Figure 3 shows the all-assigned mean SCAS trajectory by classroom with dates and post-intervention intervals; Figure 4 shows the corresponding distributions of baseline-to-follow-up reductions.

As shown in Table 4, the mean between-classroom difference in SCAS reduction was 2.98 points at 1–2 weeks, 3.79 points at 5–6 weeks, 5.09 points at 8–9 weeks, and 4.46 points at 10–11 weeks post-intervention. The two-sided Welch result at 8–9 weeks was nominally *p* = 0.050, but its FDR-adjusted value was q = 0.150. No Mann–Whitney or Welch comparison survived correction across the four follow-ups.

As summarized in Table 5, baseline-adjusted all-assigned ANCOVA estimates showed the same descriptive pattern. At 8–9 weeks, the adjusted classroom coefficient was −5.14 points (95% CI [−10.25, −0.04]) with nominal HC3 two-sided *p* = 0.048, but q = 0.135 after correction across the four follow-ups. The other estimates were smaller and statistically uncertain; no principal ANCOVA result survived FDR correction.

The secondary attendance-restricted sensitivity analysis included 52 students (Supplementary Table S5). At 8–9 weeks, its adjusted classroom coefficient was −5.30 points (95% CI [−10.69, 0.09]; HC3 two-sided *p* = 0.054; q = 0.138), compared with −5.14 points (95% CI [−10.25, −0.04]; *p* = 0.048; q = 0.135) in the all-assigned analysis. Thus, two similar point estimates fell on opposite sides of the unadjusted *p* = 0.05 threshold, but neither survived multiplicity correction. This threshold crossing illustrates sensitivity to modest changes in analytical inclusion rather than a substantively different result.

### 3.3. Baseline Anxiety Moderation and Threshold Analyses

Attendance-restricted, post hoc threshold analyses showed larger descriptive between-classroom differences among students with higher baseline SCAS scores, but no two-sided test survived FDR correction across the 21-test family (minimum q = 0.087; Supplementary Table S1). The thresholds were nested, subgroup sizes became very small, and no diagnostic criterion was applied; these analyses cannot define clinical cutoffs, caseness, benefit, or treatment responsiveness.

### 3.4. Acute POMS-A Responses

POMS-A outcomes were available only for the intervention classroom and were interpreted as acute within-classroom mood descriptions rather than controlled treatment effects. Using all available observations and two-sided tests, the POMS-TMD change was negligible during FT1, modest during FT2, largest during FT3 (*n* = 21; mean improvement = 9.71; *p* = 0.000106; q = 0.00218; dz = 1.29), and smaller during FT4 (*n* = 21; mean improvement = 4.00; *p* = 0.021; q = 0.076). Thus, only the third scheduled-session TMD result survived correction across the full 28 session-by-domain family. Calendar session, weather, attendance order, and repeated exposure were confounded. Table 6 reports the session dates and results.

The attended-order analysis recoded each student’s first, second, third, and fourth attended forest therapy experience irrespective of calendar session. POMS-TMD improvement was small at the first attended session, larger but statistically uncertain at the second, and greatest at the third (*n* = 24; mean improvement = 6.38; *p* = 0.00101; q = 0.0283; dz = 0.78). The fourth-attended-session estimate was based on only 11 students and did not survive correction. Table 7 reports the attended order and the contributing dated sessions.

### 3.5. Exploratory Associations Between Acute and Longitudinal Outcomes

To explore whether within-session affective changes were associated with longer-term changes in persistent anxiety symptoms, acute POMS improvements in the intervention classroom were correlated with SCAS reductions from baseline to follow-up. For each student, both the maximum and the mean acute improvements across attended forest-therapy sessions were computed for anxiety (POMS Tension) and total mood disturbance (POMS-TMD). These variables were then correlated with SCAS1-to-SCAS3 and SCAS1-to-SCAS4 reductions. These exploratory correlations used the attendance-restricted intervention subset (*n* = 24; at least three sessions attended).

Exploratory correlations did not support the expected positive association between acute POMS improvements and longitudinal SCAS reductions. After FDR correction across all 16 correlations, inverse POMS-TMD associations remained for maximum TMD improvement with SCAS1-to-SCAS3 reduction by Spearman correlation (q = 0.026) and for maximum and mean TMD improvement with SCAS1-to-SCAS4 reduction (q = 0.003–0.008 across Pearson and Spearman estimates). No POMS-Tension correlation survived correction. These post hoc, intervention-only associations identify no mechanism and remain hypothesis-generating (Supplementary Table S2).

### 3.6. Exploratory School-Performance Outcomes

All-assigned exploratory school-performance analyses included 53 students with complete school-record data. In ANCOVA models predicting Q2 scores adjusted for Q1 scores, the intervention-classroom coefficient was −0.113 for the 12-subject mean (*p* = 0.127, q = 0.169), −0.158 for humanities (*p* = 0.054, q = 0.107), and −0.142 for STEM (*p* = 0.438, q = 0.438). Adjusted conduct/behavior was lower in the intervention classroom (coefficient = −0.462, 95% CI [−0.797, −0.126]; *p* = 0.00698; q = 0.0279). This discordant result cannot be attributed to the program and may reflect pre-existing classroom, teacher-rating, or other educational differences (Supplementary Table S3).

### 3.7. Cross-Sectional Post-Intervention Nature-Questionnaire Descriptors

Because no baseline nature measure was available, final-questionnaire comparisons are reported only as all-assigned cross-sectional post-intervention descriptors and cannot indicate intervention-related change. The intervention classroom had descriptively higher nature-exposure and visit-frequency values, but none survived correction (q = 0.133–0.286). CNS scores were lower in the intervention classroom (difference = −0.443; *p* = 0.000970; q = 0.00485), while retrospective perceived change was similar (difference = 0.033; *p* = 0.924; q = 0.924). The multiplicity-adjusted CNS difference may be pre-existing and reinforces, rather than resolves, classroom-level confounding (Supplementary Table S4).

## 4. Discussion

### 4.1. Principal Findings

In this two-classroom pilot, the four-session program was delivered as planned during ordinary school hours, documenting operational feasibility in this specific setting. The intervention classroom showed descriptively larger SCAS reductions at each follow-up, but none of the four principal baseline-adjusted comparisons survived multiplicity correction. Because allocation was nonrandomized and condition was perfectly confounded with classroom, these observations are preliminary between-classroom signals and do not identify the program as the cause.

Attendance-restricted exploratory threshold analyses showed greater descriptive separation at some higher baseline SCAS values, but no two-sided test survived FDR correction. The sample was not selected for elevated anxiety, the thresholds were nested and post hoc, and subgroup sizes were small. The pattern cannot be used to infer clinical benefit, define caseness, or establish differential treatment response.

The results should therefore be interpreted as feasibility observations and hypothesis-generating signals rather than evidence of efficacy. The all-assigned and attendance-restricted SCAS estimates were similar, yet the nominal 8–9-week *p*-value shifted from 0.048 to 0.054 with the analytical set; neither result survived FDR correction. Two discordant exploratory findings—lower adjusted conduct/behavior and lower post-intervention CNS in the intervention classroom—further argue against selectively emphasizing favorable outcomes and reinforce the central classroom-confounding limitation.

### 4.2. From Acute Affective Responses to Longer-Term Anxiety-Symptom Trajectories

Acute POMS-A changes were clearest during the third scheduled or attended session, but POMS-A was not administered in the control classroom. Repeated questionnaire completion, relief from ordinary lessons, peer interaction, therapist attention, expectancy, weather, calendar timing, prior attendance, and regression toward the mean remain plausible explanations. The data cannot attribute the acute pattern specifically to the forest environment or any component of the bundled program.

Evidence on acute responses across repeated nature-therapy sessions in adolescents is limited. In a three-week forest-bathing program for 16–18 year olds, acute improvements in well-being were observed after individual sessions, while broader mental-health risk declined only after repeated exposure (Keller et al., 2024). That study lacked a control group and follow-up assessments, limiting conclusions about persistence.

Joint assessment of acute affect and later anxiety-symptom trajectories is a conceptual contribution of this pilot, but the two outcomes have different timescales and evidentiary meanings. Acute changes need not accumulate into persistent symptom change, which would require maintenance or generalization beyond a session. The post hoc individual-level analyses found no positive association between larger acute POMS-A improvements and larger subsequent SCAS reductions. Acute and longitudinal outcomes should therefore be regarded as distinct, and candidate pathways such as attention, arousal, perceived stress, emotion regulation, or co-regulation remain unmeasured.

Prior studies provide limited evidence that some psychological or physiological effects of forest and nature-based interventions can persist beyond the intervention period. Reductions in salivary cortisol (Sung et al., 2012) and changes in immune-related outcomes (Dai et al., 2025; Li, 2010) have sometimes been reported after forest exposure, although these mechanisms were not measured in the present study. Evidence for sustained anxiety or mood outcomes remains comparatively sparse (Qin et al., 2026). Four weekly self-guided forest-bathing sessions maintained gains in positive affect and well-being for approximately one month (Markwell & Gladwin, 2020), while a 12-week nature-walking program in adult women showed residual well-being benefits at later follow-ups (Chauvenet et al., 2025). Other uncontrolled or multicomponent studies have reported anxiety or depression benefits from one to several weeks after intervention (Chun et al., 2023), and a fibromyalgia trial suggested that immediate state-anxiety relief and change in more persistent distress may require different intervention doses (Serrat et al., 2020). These heterogeneous adult findings provide context but cannot establish the duration or mechanism of effects in adolescents.

### 4.3. Intervention Components and Interpretive Boundaries

The intervention bundled slow walking, multisensory invitations, therapist-guided pacing, and participation with a familiar peer group. Prior research has distinguished guided from self-guided delivery (Rivieccio et al., 2025), but the present design did not isolate any component. Multisensory attention, therapist-guided pacing, attentional restoration, emotion regulation, perceived stress recovery, and group co-regulation were not directly measured. They are candidate pathways for future mediation studies, not mechanisms supported by these data. Acute–longitudinal correlations identify post hoc associations rather than processes, while academic and post-intervention nature-questionnaire measures cannot be interpreted as mediators.

The program represents only one form and dose of nature-based intervention: four approximately 3 h sessions over four weeks, emphasizing slow, low-exertion movement and guided multisensory attention. Gardening or horticultural programs additionally emphasize task mastery and stewardship; mindfulness in green settings centers formal contemplative practice; and green exercise includes a larger physical-activity component. The present study neither compared these approaches nor established the four-session schedule as optimal. Comparative studies should match duration, outdoor exposure, group interaction, facilitator attention, and expectancy while varying the intervention component.

### 4.4. Implications for Adolescent Mental Health and School-Based Prevention

This study examined delivery by one school psychotherapist supported by one school psychologist after study-protocol training. Delivery of four sessions by this particular team does not establish minimum qualifications, transferability to teachers or non-clinical staff, scalability, or readiness for routine implementation. The descriptive findings provide parameters for a larger trial but do not establish effectiveness, clinical benefit, or the specific contribution of forest setting, professional guidance, classmates, movement, expectancy, or time away from lessons.

The outdoor, non-pharmacological, and low-physical-intensity format may be acceptable to some students, but acceptability, stigma, expectancy, fidelity, adverse events, and implementation outcomes were not formally measured. Forest therapy should complement rather than replace diagnostic assessment or evidence-based care. Routine school implementation would require defined facilitator competencies and scope of practice, protocol training and supervision, outdoor and group risk management, voluntary participation and withdrawal procedures, confidential review of marked or worsening anxiety symptoms, safeguarding and escalation procedures, and referral pathways to school and local health services.

Connectedness to nature may be relevant to mental health, pro-environmental behavior, and social relationships (Chapman et al., 2025; Madera et al., 2025). In the all-assigned analysis, post-intervention CNS scores were lower in the intervention classroom than in the control classroom (difference = −0.443; q = 0.00485), while retrospective perceived change was similar. Because no baseline nature measure was collected, these are cross-sectional descriptors that may reflect pre-existing differences and cannot be interpreted as intervention-related change or mediation. Their unfavorable direction is reported without selective minimization and reinforces the classroom-confounding concern.

### 4.5. Limitations

The principal limitation is the one-classroom-per-condition design. The effective number of independently assigned units was two, and condition was perfectly confounded with classroom. Student-level HC3 estimates and exploratory longitudinal models cannot estimate unobserved between-classroom variability or convert the design into a cluster-level test. Allocation was nonrandomized, the sample was small, no formal a priori power analysis was conducted, and the study was not preregistered. The principal all-assigned cohort was 94.4% female (51/54) and included only three male students, and school records did not separately capture gender-diverse identities; generalizability to male and gender-diverse adolescents is therefore severely limited. The attendance-restricted sensitivity analysis does not eliminate adherence or classroom-level confounding, and its nominal 8–9-week *p*-value crossed 0.05 despite a point estimate similar to the principal result.

Participants were not selected for elevated anxiety, and SCAS-C is a symptom questionnaire rather than a diagnostic instrument. Psychometric evidence is stronger through age 17 than at age 18. Although no obvious total-score ceiling was observed, this does not establish validity or measurement invariance at age 18. No complementary late-adolescent anxiety measure, diagnostic interview, depressive-symptom assessment, information on learning or neurodevelopmental conditions, functional-impairment measure, medication, psychotherapy, or concurrent-treatment data were available. The study therefore cannot characterize comorbidity or clinical efficacy.

POMS-A was administered only in the intervention classroom, and the usual-activities comparator did not isolate forest exposure, peer interaction, therapist contact, guided sensory activity, physical movement, expectancy, relief from lessons, repeated testing, weather, or regression toward the mean. Participants knew their classroom allocation and participation; facilitators and questionnaire administrators were not blinded. The principal mental-health outcome relied exclusively on repeated student self-report, with no parent-, teacher-, clinician-, or independently blinded anxiety assessment, increasing susceptibility to expectancy, demand, and reporting effects. No formal fidelity checklist, independent observer, recording, adherence score, or inter-rater assessment was used. No physiological, attentional, emotion-regulation, or stress-recovery process was measured. The post-intervention nature questionnaire lacked a baseline. Although FDR correction was applied to defined two-sided analytical families, the analyses were not preregistered, several exploratory families involved nested or correlated measures, and subgroup sizes were small. A small amount of item-level imputation was used before scale-score calculation. Although no complete assessment occasion, participant-level scale total, or change score was imputed as a unit, the inclusion of estimated item responses may have influenced individual scores and remains a source of uncertainty in this small pilot.

### 4.6. Future Research

Future studies should distinguish universal school-based prevention from clinically targeted intervention. Replicated, preregistered cluster-randomized trials should include multiple independently assigned classrooms per condition across several schools, an a priori sample-size calculation, acute measures in every condition, and an active comparator matched for time, movement, group interaction, expectancy, and facilitator attention. A prespecified facilitator manual, competence-based training, supervision, session checklist, and independent or recorded fidelity assessment should accompany formal evaluation of acceptability, adverse events, referrals, and implementation outcomes. Safeguarding procedures should specify a confidential review of marked or worsening symptoms and referral or escalation pathways to school and health services. Clinically oriented studies should prespecify symptom or diagnostic eligibility, use measures validated across the full age range with multi-informant or clinician assessment where appropriate, and assess depressive symptoms, relevant learning or neurodevelopmental conditions, functional impairment, and concurrent care. Longer follow-ups, baseline and follow-up nature measures, and direct physiological, attentional, or emotion-regulation measures with appropriate temporal sequencing would be required to test duration and candidate mechanisms.

## 5. Conclusions

In this two-classroom school pilot, four therapist-guided forest therapy sessions were operationally delivered as planned in this specific setting. The intervention classroom showed descriptively larger SCAS reductions than the usual-activities classroom, with the largest adjusted separation at 8–9 weeks, but no principal comparison survived multiplicity correction, and the nominal *p*-value was sensitive to analytical inclusion. Acute POMS-A change was clearest during the third session but lacked a control-classroom comparator. Lower adjusted conduct/behavior and lower post-intervention connectedness to nature in the intervention classroom are discordant exploratory findings that further underscore classroom confounding. Because participants were not clinically selected, allocation was nonrandomized, only one classroom represented each condition, outcomes were nonblinded self-reports, and no active comparator was included, the observations cannot be attributed to forest therapy and do not establish clinical efficacy or intervention-specific effects. They are feasibility observations and hypothesis-generating signals for larger, preregistered, adequately replicated cluster-randomized studies.

## Figures and Tables

**Table 1:** Weather and discomfort data for each forest therapy session.

Session ID	Date	Temperature ^a^ (°C)	Relative Humidity ^b^ (%)	Discomfort Index ^c^	Cloudiness ^d^ (/8)	Rainfall ^e^	Wind ^f^ (m/s)
FT1	2 March 2026	12.0–15.5	80–65	12.3–15.3	0	Absent	2.0
FT2	12 March 2026	12.0–13.5	45–45	12.8–13.8	8	Weak, intermittent	3.5
FT3	18 March 2026	11.0–13.5	40–50	12.2–13.8	0	Absent	11.0
FT4	26 March 2026	10.0–13.0	77–43	10.6–13.5	5	Absent	10.0

^a^ Air-temperature range measured at 2 m from the beginning to the end of each forest therapy session; ^b^ relative-humidity range measured at 2 m from the beginning to the end of each session; ^c^ discomfort-index range from the beginning to the end of each session; ^d^ cloud cover in eighths, visually estimated by an expert meteorologist; ^e^ rainfall during each session; ^f^ mean wind speed measured at 10 m during each session.

**Table 2:** Attendance and baseline anxiety-symptom levels in the principal all-classroom-assigned cohort.

Classroom	*n*	Female	Male	Attendance	Baseline SCAS Mean ± SD
4AU (intervention)	26	25	1	11 attended 4 sessions; 13 attended 3; 1 attended 2; 1 attended 0	41.88 ± 15.04
4BU (control)	28	26	2	No forest therapy sessions	42.54 ± 16.28

**Table 3:** SCAS raw scores and reductions by classroom in the principal all-assigned cohort. Follow-up intervals are relative to the final forest therapy session on 26 March 2026. SD = standard deviation; IQR = interquartile range (Q3–Q1).

Classroom	*n*	Variable	Mean ± SD	Median	IQR	Min	Max
4AU	26	Baseline (SCAS1)	41.88 ± 15.04	38.50	25.75	16.00	70.00
		1–2 weeks post (SCAS2)	36.65 ± 14.93	33.50	27.00	10.00	61.00
		5–6 weeks post (SCAS3)	35.88 ± 14.45	32.50	24.75	14.00	61.00
		8–9 weeks post (SCAS4)	38.15 ± 15.06	35.50	23.50	12.00	68.00
		10–11 weeks post (SCAS5)	37.42 ± 17.16	34.00	26.00	13.00	71.00
		Reduction at 1–2 weeks post	5.23 ± 8.76	3.50	9.75	−6.00	28.00
		Reduction at 5–6 weeks post	6.00 ± 9.67	3.00	9.75	−6.00	31.00
		Reduction at 8–9 weeks post	3.73 ± 10.43	2.00	11.75	−10.00	29.00
		Reduction at 10–11 weeks post	4.46 ± 11.39	4.00	14.25	−12.00	32.00
4BU	28	Baseline (SCAS1)	42.54 ± 16.28	40.50	21.25	16.00	79.00
		1–2 weeks post (SCAS2)	40.29 ± 16.32	37.00	24.50	15.00	81.00
		5–6 weeks post (SCAS3)	40.32 ± 18.15	35.00	23.75	17.00	89.00
		8–9 weeks post (SCAS4)	43.89 ± 18.54	42.50	31.25	18.00	86.00
		10–11 weeks post (SCAS5)	42.54 ± 18.90	41.00	28.75	16.00	83.00
		Reduction at 1–2 weeks post	2.25 ± 5.75	1.00	7.50	−7.00	13.00
		Reduction at 5–6 weeks post	2.21 ± 7.46	3.00	10.50	−15.00	15.00
		Reduction at 8–9 weeks post	−1.36 ± 7.84	0.00	10.00	−21.00	15.00
		Reduction at 10–11 weeks post	0.00 ± 9.43	2.50	14.25	−27.00	15.00

**Table 4:** Two-sided between-classroom comparisons of SCAS reductions in the principal all-assigned cohort. Difference = intervention classroom minus control classroom; 95% CIs are two-sided Welch confidence intervals. Benjamini–Hochberg q-values were adjusted across the four follow-ups separately for each method.

Follow-Up	4AU Mean Reduction	4BU Mean Reduction	Difference (95% CI)	Mann–Whitney *p* (FDR q) (Two-Sided)	Welch *p* (FDR q) (Two-Sided)	Cohen’s d
1–2 weeks post (SCAS2)	5.23	2.25	2.98 [−1.12, 7.08]	0.245 (0.278)	0.150 (0.150)	0.41
5–6 weeks post (SCAS3)	6.00	2.21	3.79 [−0.97, 8.54]	0.278 (0.278)	0.116 (0.150)	0.44
8–9 weeks post (SCAS4)	3.73	−1.36	5.09 [0.00, 10.17]	0.143 (0.278)	0.050 (0.150)	0.55
10–11 weeks post (SCAS5)	4.46	0.00	4.46 [−1.28, 10.20]	0.267 (0.278)	0.125 (0.150)	0.43

**Table 5:** Principal all-assigned ANCOVA models adjusting follow-up SCAS for baseline SCAS. Adjusted classroom coefficients compare the intervention classroom with the control classroom; negative coefficients favor the intervention classroom descriptively. Two-sided 95% CIs and *p*-values use HC3 robust standard errors; Benjamini–Hochberg q-values were adjusted across the four follow-ups.

Follow-Up	*n*	Adjusted Classroom Coefficient (95% CI)	HC3 *p* (Two-Sided)	FDR q	R^2^
1–2 weeks post (SCAS2)	54	−3.05 [−7.05, 0.95]	0.135	0.135	0.795
5–6 weeks post (SCAS3)	54	−3.85 [−8.59, 0.89]	0.112	0.135	0.742
8–9 weeks post (SCAS4)	54	−5.14 [−10.25, −0.04]	0.048	0.135	0.722
10–11 weeks post (SCAS5)	54	−4.50 [−10.33, 1.33]	0.131	0.135	0.677

**Table 6:** Acute changes in total mood disturbance (POMS-TMD) by scheduled session and date using all available observations. Positive values indicate improvement (pre minus post). Wilcoxon *p*-values are two-sided; FDR q-values are Benjamini–Hochberg adjusted across the full 28 scheduled-session-by-domain family. dz = standardized paired effect size.

Session	*n*	Mean TMD PRE	Mean TMD POST	Mean Improvement	Wilcoxon *p* (Two-Sided)	FDR q	dz
FT1 (2 March)	22	9.36	9.23	0.14	0.875	0.940	0.02
FT2 (12 March)	21	20.10	17.71	2.38	0.533	0.648	0.23
FT3 (18 March)	21	15.33	5.62	9.71	0.00011	0.0022	1.29
FT4 (26 March)	21	16.86	12.86	4.00	0.021	0.076	0.60

**Table 7:** Acute changes in total mood disturbance (POMS-TMD) by order of sessions attended, using all available observations. Positive values indicate improvement (pre minus post). Wilcoxon *p*-values are two-sided; FDR q-values are Benjamini–Hochberg adjusted across the full 28 attended-order-by-domain family. dz = standardized paired effect size.

Attended Order	*n*	Calendar Sessions	Mean TMD PRE	Mean TMD POST	Mean Improvement	Wilcoxon *p* (Two-Sided)	FDR q	dz
First attended	25	FT1: 22; FT2: 2; FT3: 1	12.28	11.52	0.76	0.586	0.690	0.09
Second attended	25	FT2: 19; FT3: 5; FT4: 1	19.24	14.84	4.40	0.063	0.148	0.44
Third attended	24	FT3: 15; FT4: 9	14.00	7.62	6.38	0.0010	0.028	0.78
Fourth attended	11	FT4: 11	16.36	11.00	5.36	0.041	0.115	0.71

## Data Availability

The de-identified analysis-ready dataset, dataset metadata, and Python analysis script are publicly available in Zenodo at https://doi.org/10.5281/zenodo.21286542. Item-level questionnaire data and more granular school-grade records are not shared because the study involved minors; only de-identified and analysis-ready variables compatible with privacy, institutional, and consent constraints are provided.

## Supplementary Materials

The following supporting information can be downloaded at https://www.mdpi.com/article/10.3390/bs16091647/s1: Table S1, Attendance-restricted, post hoc baseline-SCAS threshold analyses of change from SCAS1 (baseline) to SCAS4 (57–60 days after the final scheduled session). The analytical set includes 4AU students attending at least three sessions and all 4BU students. Positive reduction values indicate lower SCAS scores at follow-up. Thresholds are nested exploratory stratification cut points, not validated clinical cutoffs. All Mann–Whitney, Welch, and HC3 ANCOVA *p*-values are two-sided and followed by Benjamini–Hochberg false-discovery-rate q-values as *p* (q); the correction family comprises the 21 tests shown. Negative ANCOVA coefficients favor 4AU descriptively; Table S2, Hypothesis-generating associations in the attendance-restricted 4AU subset (*n* = 24; at least three sessions attended) between acute Profile of Mood States improvements and subsequent SCAS reductions. SCAS3 was assessed 34–39 days and SCAS4 57–60 days after the final scheduled session. Positive ΔANX and ΔTMD values indicate acute improvement; positive SCAS reduction values indicate lower follow-up scores than at baseline. Pearson and Spearman *p*-values are two-sided and followed by Benjamini–Hochberg q-values as *p* (q); the correction family comprises all 16 correlation tests shown. Associations are exploratory and do not establish a causal pathway; Table S3, All-assigned exploratory HC3-robust ANCOVA models predicting second-period (Q2; end-of-school assessment) school outcomes from classroom and first-period (Q1; pre-intervention) values. Fifty-three of 54 students had complete school-record data. Positive adjusted classroom coefficients favor 4AU. Two-sided 95% CIs are shown; *p*-values are followed by Benjamini–Hochberg q-values as *p* (q), with correction across the four models; Table S4, All-assigned cross-sectional post-intervention nature-questionnaire descriptors by classroom (4AU *n* = 26; 4BU *n* = 28). These measures were administered once during the final follow-up period and do not estimate within-person change attributable to forest therapy. The exposure questions referred to April–May 2026. The nature-exposure index ranges from 0 to 100; CNS denotes the Connectedness to Nature Scale mean. Perceived change is a retrospective self-rating, not a prospectively measured change score. Differences and signed Cohen’s d are 4AU minus 4BU. Two-sided Mann–Whitney *p*-values are followed by Benjamini–Hochberg q-values as *p* (q), with correction across the five descriptors. Classroom differences are exploratory and should not be interpreted causally; Table S5, Attendance-restricted sensitivity analyses including 24 intervention-classroom students who attended at least three forest therapy sessions and all 28 control-classroom students. SCAS reductions are baseline minus follow-up; positive values indicate improvement, and differences are 4AU minus 4BU. Follow-up labels give the observed range of days after the final scheduled session. ANCOVA models predict follow-up SCAS from baseline SCAS and classroom; negative coefficients favor 4AU descriptively. All *p*-values are two-sided and followed by Benjamini–Hochberg q-values; correction was applied across the four follow-ups separately within each inferential method.

## Abbreviations

The following abbreviations are used in this manuscript:

ANCOVA	Analysis of covariance
CI	Confidence interval
CNS	Connectedness to Nature Scale
DI	Discomfort index
FDR	False discovery rate
FSL	Formazione Scuola-Lavoro (school-to-work training program)
FT	Forest therapy session
GEE	Generalized estimating equation
HC3	Heteroskedasticity-consistent covariance estimator, type 3
IQR	Interquartile range
NBI	Nature-based intervention
POMS-A	Profile of Mood States—Adolescents
POMS Tension	POMS-A tension subscale
POMS-TMD	POMS-A total mood disturbance
Q1	First grading period
Q2	Second grading period
SCAS	Spence Children’s Anxiety Scale
SCAS-C	Spence Children’s Anxiety Scale—Child version
SD	Standard deviation
SEM	Standard error of the mean
STEM	Science, technology, engineering, and mathematics
TMD	Total mood disturbance
