# Supplementary material for: Therapist-Guided Forest Therapy in Adolescents: Operational Feasibility and Anxiety-Symptom Signals from a Two-Classroom Quasi-Experimental Pilot Study
Source: Behav Sci (Basel). 2026 Sep 14;16(9):1647. doi: 10.3390/bs16091647 (PMC13603247; doi:10.3390/bs16091647)
Supplement: Supplementary file 1 [file behavsci-16-01647-s001.zip › behavsci-4467950-supplementary.pdf]

Article

Therapist-Guided Forest Therapy in Adolescents: Operational Feasibility and Anxiety-Symptom Signals from a Two-Classroom Quasi-Experimental Pilot Study

Francesco Meneguzzo [1,2,*] and Federica Zabini [1]

[1] Institute of Bioeconomy, National Research Council, 10 Via Madonna del Piano, I-50019 Sesto Fiorentino, Italy; federica.zabini@cnr.it
[2] Consorzio LaMMA, Via Madonna del Piano 10, I-50019 Sesto Fiorentino, Italy
[*] Correspondence: francesco.meneguzzo@cnr.it; Tel.: +39-392-9850002

Supplementary materials

Table S1. Attendance-restricted, post hoc baseline-SCAS threshold analyses of change from SCAS1 (baseline) to SCAS4 (57–60 days after the final scheduled session). The analytical set includes 4AU students attending at least three sessions and all 4BU students. Positive reduction values indicate lower SCAS scores at follow-up. Thresholds are nested exploratory stratification cut points, not validated clinical cutoffs. All Mann–Whitney, Welch, and HC3 ANCOVA p-values are two-sided and followed by Benjamini–Hochberg false-discovery-rate q-values as p (q); the correction family comprises the 21 tests shown. Negative ANCOVA coefficients favor 4AU descriptively.

SCAS1 threshold	n 4AU	n 4BU	4AU mean reduction	4BU mean reduction	Difference	Mann–Whitney two-sided p (q)	Welch two-sided p (q)	ANCOVA coefficient	HC3 two-sided p (q)
≥30	17	22	5.59	−0.95	6.54	0.078 (0.091)	0.044 (0.087)	−6.54	0.043 (0.087)
≥35	13	18	7.77	−1.17	8.94	0.022 (0.087)	0.022 (0.087)	−9.17	0.013 (0.087)
≥40	10	15	7.10	−1.47	8.57	0.075 (0.091)	0.078 (0.091)	−8.97	0.060 (0.087)
≥45	10	12	7.10	−3.00	10.10	0.047 (0.087)	0.041 (0.087)	−10.07	0.036 (0.087)
≥50	10	8	7.10	−2.88	9.97	0.055 (0.087)	0.041 (0.087)	−10.24	0.024 (0.087)
≥55	8	7	7.75	−2.43	10.18	0.115 (0.120)	0.088 (0.097)	−9.80	0.062 (0.087)
≥60	3	5	14.00	−2.60	16.60	0.036 (0.087)	0.136 (0.136)	−15.96	0.062 (0.087)

Table S2. Hypothesis-generating associations in the attendance-restricted 4AU subset (n = 24; at least three sessions attended) between acute Profile of Mood States improvements and subsequent SCAS reductions. SCAS3 was assessed 34–39 days and SCAS4 57–60 days after the final scheduled session. Positive ΔANX and ΔTMD values indicate acute improvement; positive SCAS reduction values indicate lower follow-up scores than at baseline. Pearson and Spearman p-values are two-sided and followed by Benjamini–Hochberg q-values as p (q); the correction family comprises all 16 correlation tests shown. Associations are exploratory and do not establish a causal pathway.

Acute POMS predictor	Longitudinal SCAS outcome	n	Pearson r	R^2	Pearson p (q)	Spearman ρ	Spearman p (q)
Max ΔANX	SCAS1→SCAS3 (34–39 days)	24	−0.236	0.056	0.267 (0.285)	−0.285	0.177 (0.214)
Max ΔANX	SCAS1→SCAS4 (57–60 days)	24	−0.350	0.122	0.094 (0.125)	−0.391	0.059 (0.117)
Mean ΔANX	SCAS1→SCAS3 (34–39 days)	24	−0.223	0.050	0.295 (0.295)	−0.279	0.187 (0.214)
Mean ΔANX	SCAS1→SCAS4 (57–60 days)	24	−0.370	0.137	0.075 (0.119)	−0.426	0.038 (0.087)
Max ΔTMD	SCAS1→SCAS3 (34–39 days)	24	−0.455	0.207	0.025 (0.068)	−0.527	0.008 (0.026)
Max ΔTMD	SCAS1→SCAS4 (57–60 days)	24	−0.660	0.436	<0.001 (0.003)	−0.675	<0.001 (0.003)
Mean ΔTMD	SCAS1→SCAS3 (34–39 days)	24	−0.362	0.131	0.082 (0.119)	−0.370	0.075 (0.119)
Mean ΔTMD	SCAS1→SCAS4 (57–60 days)	24	−0.598	0.358	0.002 (0.008)	−0.648	<0.001 (0.003)

Table S3. All-assigned exploratory HC3-robust ANCOVA models predicting second-period (Q2; end-of-school assessment) school outcomes from classroom and first-period (Q1; pre-intervention) values. Fifty-three of 54 students had complete school-record data. Positive adjusted classroom coefficients favor 4AU. Two-sided 95% CIs are shown; p-values are followed by Benjamini–Hochberg q-values as p (q), with correction across the four models.

Outcome	Adjusted 4AU coefficient (95% CI) for Q2	HC3 p (q) (two-sided)	R^2
12-subject academic mean	−0.113 [−0.258, 0.032]	0.127 (0.169)	0.787
Humanities mean	−0.158 [−0.319, 0.002]	0.054 (0.107)	0.768
STEM mean	−0.142 [−0.500, 0.216]	0.438 (0.438)	0.588
Conduct/behavior	−0.462 [−0.797, −0.126]	0.007 (0.028)	0.350

Table S4. All-assigned cross-sectional post-intervention nature-questionnaire descriptors by classroom (4AU n = 26; 4BU n = 28). These measures were administered once during the final follow-up period and do not estimate within-person change attributable to forest therapy. The exposure questions referred to April–May 2026. The nature-exposure index ranges from 0 to 100; CNS denotes the Connectedness to Nature Scale mean. Perceived change is a retrospective self-rating, not a prospectively measured change score. Differences and signed Cohen's d are 4AU minus 4BU. Two-sided Mann–Whitney p-values are followed by Benjamini–Hochberg q-values as p (q), with correction across the five descriptors. Classroom differences are exploratory and should not be interpreted causally.

Descriptor	4AU mean	4BU mean	Difference (4AU−4BU)	Mann–Whitney p (q) (two-sided)	Signed Cohen's d (4AU−4BU)
Nature-exposure index	57.23	51.11	6.12	0.229 (0.286)	0.32
Natural-environment visit frequency	3.38	2.79	0.60	0.053 (0.133)	0.52
Forest/pinewood visit frequency	2.46	2.00	0.46	0.090 (0.150)	0.38
Connectedness to Nature Scale mean	2.98	3.42	−0.44	0.0010 (0.0049)	−0.88
Perceived change in relationship with nature	3.33	3.30	0.03	0.924 (0.924)	0.06

Table S5. Attendance-restricted sensitivity analyses including 24 intervention-classroom students who attended at least three forest therapy sessions and all 28 control-classroom students. SCAS reductions are baseline minus follow-up; positive values indicate improvement, and differences are 4AU minus 4BU. Follow-up labels give the observed range of days after the final scheduled session. ANCOVA models predict follow-up SCAS from baseline SCAS and classroom; negative coefficients favor 4AU descriptively. All p-values are two-sided and followed by Benjamini–Hochberg q-values; correction was applied across the four follow-ups separately within each inferential method.

SCAS interval (days after final session)	n 4AU	n 4BU	4AU mean reduction	4BU mean reduction	Difference (95% CI)	MW U two-sided p (q)	Welch two-sided p (q)	Cohen's d	ANCOVA coefficient (95% CI)	HC3 two-sided p (q)	R^2
SCAS1→2 (5–13 days)	24	28	5.33	2.25	3.08 [−1.29, 7.45]	0.247 (0.329)	0.161 (0.172)	0.41	−3.17 [−7.41, 1.07]	0.143 (0.179)	0.794
SCAS1→3 (34–39 days)	24	28	6.67	2.21	4.45 [−0.46, 9.37]	0.171 (0.329)	0.075 (0.150)	0.52	−4.53 [−9.41, 0.35]	0.069 (0.138)	0.750
SCAS1→4 (57–60 days)	24	28	3.88	−1.36	5.23 [−0.15, 10.62]	0.144 (0.329)	0.057 (0.150)	0.56	−5.30 [−10.69, 0.09]	0.054 (0.138)	0.721
SCAS1→5 (70–78 days)	24	28	4.17	0.00	4.17 [−1.88, 10.21]	0.398 (0.398)	0.172 (0.172)	0.39	−4.21 [−10.35, 1.92]	0.179 (0.179)	0.677

Interpretive note: At SCAS4, the attendance-restricted adjusted coefficient was −5.30 (95% CI [−10.69, 0.09]; HC3 $p = 0.054$; $q = 0.138$), compared with −5.14 (95% CI [−10.25, −0.04]; $p = 0.048$; $q = 0.135$) in the principal all-assigned analysis. The similar estimates crossed the unadjusted $p = 0.05$ threshold in opposite directions, but neither survived multiplicity correction. No attendance-restricted sensitivity contrast had $q < 0.05$. These analyses do not remove classroom confounding or establish a dose response.
